# Frequency-Resolved Functional Connectivity: Role of Delay and the Strength of Connections

**DOI:** 10.3389/fncir.2021.608655

**Published:** 2021-03-24

**Authors:** Abolfazl Ziaeemehr, Alireza Valizadeh

**Affiliations:** ^1^Department of Physics, Institute of Advanced Studies in Basic Sciences, Zanjan, Iran; ^2^School of Biological Sciences, Institute for Research in Fundamental Sciences, Tehran, Iran

**Keywords:** functional connectivity, functional network, connectome, transmission delay, brain oscillation, correlation matrix, hierarchical clustering

## Abstract

The brain functional network extracted from the BOLD signals reveals the correlated activity of the different brain regions, which is hypothesized to underlie the integration of the information across functionally specialized areas. Functional networks are not static and change over time and in different brain states, enabling the nervous system to engage and disengage different local areas in specific tasks on demand. Due to the low temporal resolution, however, BOLD signals do not allow the exploration of spectral properties of the brain dynamics over different frequency bands which are known to be important in cognitive processes. Recent studies using imaging tools with a high temporal resolution has made it possible to explore the correlation between the regions at multiple frequency bands. These studies introduce the frequency as a new dimension over which the functional networks change, enabling brain networks to transmit multiplex of information at any time. In this computational study, we explore the functional connectivity at different frequency ranges and highlight the role of the distance between the nodes in their correlation. We run the generalized Kuramoto model with delayed interactions on top of the brain's connectome and show that how the transmission delay and the strength of the connections, affect the correlation between the pair of nodes over different frequency bands.

## 1. Introduction

A very prominent feature of the brain is the ability to dynamically changing the routes for communication between the brain regions when undertaking different cognitive and executive functions (Honey et al., 2007; Friston, 2011; Valdes-Sosa et al., 2011; Park et al., 2018). This is revealed by extensive studies on the pattern of statistical inter-relations between the activities of different brain regions at different brain states based on BOLD signals (Chang and Glover, 2010; Allen et al., 2014; Calhoun et al., 2014; Wang et al., 2016; Park et al., 2018). The brain functional network is defined based on the level of linear pair-wise correlation or other non-linear measures between the activities of the different regions (Gao et al., 2012; Rubchinsky et al., 2012). These correlated activities are supposed to underlie the integration of information over subsets of the whole-brain structural network, comprising different functional modules (Friston, 2002). It has been shown that, due to environmental demands and changes in the state of the brain, regions of the brain can engage in functional modules and detach from others, allowing the brain to switch between multiple tasks over time (Gonzalez-Castillo et al., 2015; Hansen et al., 2015).

Functional networks are not only defined based on the BOLD signals but also they can be constructed upon the electrophysiological data using EEG and MEG tools (Rodríguez-Rivera et al., 2006; Haufe et al., 2011). Each of the methods has its advantages and disadvantages. BOLD signals have low temporal resolution and can only capture the slow dynamics of the brain regions, but they have higher spatial resolution compared to EEG and MEG. Electrophysiological data on the other hand provides a good temporal resolution but they suffer from difficulties in source localization (Pascual-Marqui, 1999). Despite the shortcomings, the higher temporal resolution of these tools extend the studies on the functional networks to the frequency domains which were not accessible through fMRI. This frequency range spans several specific bands (including delta, alpha, beta, and gamma bands) which are hypothesized to be important in several perceptional, cognitive, and executive brain functions (Schnitzler and Gross, 2005; De Pasquale et al., 2010; Brookes et al., 2016; Tewarie et al., 2016; Li et al., 2017). For example, coherence in the gamma range is hypothesized to provide a means for controlling communication between the brain regions, according to “communication through coherence” theory (Womelsdorf and Fries, 2007; Schroeder and Lakatos, 2009; Ray and Maunsell, 2015; Bonnefond et al., 2017).

Recent studies using MEG have shown that functional networks not only change in time (De Pasquale et al., 2010) but also they are frequency-dependent and multiple functional networks are present at any given time over different frequency bands (Hillebrand et al., 2012). These observations assert that any region can simultaneously participate in multiple functional modules, acting in parallel, and exploiting the structural communication channels for multiple tasks (Brookes et al., 2011, 2016).

In this study, we question what properties of the brain structural network determine the pattern of the frequency-resolved functional network (Ziaeemehr et al., 2020a). Our focus is on the role of the Euclidean distance between the brain regions on the pairwise correlation between their activity at different frequencies. Euclidean distance between the brain regions determines the length of connecting axons (Nakagawa et al., 2014) and therefore, the delay in the transmission of the signals between them (Nakagawa et al., 2014; Petkoski et al., 2018). On the other hand, the strength of the connections defined as the number of tracts in diffusion MRI shows a negative correlation with distance (Fox et al., 2005; Honey et al., 2007). Despite the debates and possible shortcomings in the interpretation of the diffusion MRI data for the determination of the connection's strengths, other methods confirm the presence of a similar relation between the strengths of the connections and the distance between the brain regions (Ercsey-Ravasz et al., 2013; Markov et al., 2013). By the simulation of a simple model composed of phase oscillators, on top of the brain structural network and changing the natural frequency of the nodes (brain regions), we show that the mean correlation between the oscillatory activity of the brain regions decreases with frequency and anticorrelations are seen at higher frequencies, compatible with previous studies (Fox et al., 2005; Deco et al., 2009; Lewis et al., 2009; Li and Zhou, 2011). We also show that variation of the correlation with frequency is more profound for the pair with longer distances. Likewise, the variation of the correlation with distance, in general, depends on the frequency and more variation is observed at higher frequencies.

Since both the delay in the interaction and the strength of the connections are dependent on the distance, we then focus on the distinct effects of these two parameters. We show that increasing the distance, the correlations change in an almost periodic manner. The period is determined by the delay in the interaction between the nodes while the amplitude of the variations is mostly affected by the strength of the structural connections. Our results highlight the distinct role of the strength and the delay of the structural connections in the pattern of correlations between the brain regions and consequently, in the functional connectivity of brain networks.

## 2. Model and Methods

The Kuramoto model has been used to describe large-scale network synchronization (Breakspear et al., 2010; Cabral et al., 2011, 2014). Each node in the model represents the oscillatory activity of a region of interest (ROI) connected by the links which are based on the structural connections between the brain regions (Bullmore and Sporns, 2009; Van den Heuvel and Sporns, 2013). The important network parameters are strength of the connections and their delay, both are set using available data about the connections in large scale brain connectome. The generalized Kuramoto model (with delay) obeys the following dynamical equation (Yeung and Strogatz, 1999; Lee et al., 2009):

(1) $$\begin{array}{l} \dot{\theta_{i}}=\omega_{i}+\xi_{i}\left(t\right)+\frac{K}{N}\underset{j=1}{\overset{N}{\sum }}a_{ij}\sin\left[\theta_{j}\left(t-\tau_{ij}\right)-\theta_{i}\left(t\right)\right]\;, \end{array}$$

where θ_*i*_(*t*) denotes the phase of node *i* at time t, ω_*i*_ = 2πν_*i*_ is the natural angular frequency of the *i*-th oscillator. *a*_*ij*_ are the elements of the weighted adjacency matrix which are derived from structural network: *A*. 0 < *a*_*ij*_ ≤ 1 if there is a link from the node *i* to *j* with a time delay τ_*ij*_; otherwise *a*_*ij*_ = 0. The parameter *K* sets the overall coupling strength.

The initial values of θ_*i*_ are randomly drawn from a uniform distribution in the interval [0, 2π], and natural frequencies are drawn from a narrow normal distribution with a given mean as a parameter and standard deviation of 0.1. We used a small-amplitude Gaussian white noise with mean zero and standard deviation SD = 0.05. Adding noise assures that the resultant numerical solutions of Equation (1) are not spurious.

To construct functional network we use correlation index which is a measure of the degree of synchronization between any two nodes of the network, defined as σ_*ij*_ = 〈cos[θ_*i*_(*t*)−θ_*j*_(*t*)]〉. Here, 〈…〉 represents averaging over different initial conditions. Correlation index is zero for uncorrelated phases and is equal to 1 (−1) for fully correlated (anti-correlated) phases. We take σ_*ij*_ as the elements of the functional network (Arenas et al., 2006).

The system of delayed differential equations (DDE) (Equation 1) is solved numerically using adaptive Bogacki-Shampine (Flunkert, 2011) with minimum time step 0.001 ms, absolute and relative error tolerance of 10^−8^ and 10^−5^, respectively. For noisy delayed differential equations, the deterministic parts of the equations are solved using a high order method as described above. Finally, the noise is added to each step implemented via the Euler-Maruyama scheme. We discarded the first 7 s and continued the simulations for 12 s and repeated the simulations 200 times with different initial conditions and natural frequency distribution.

To quantify the similarity between the functional and structural networks, we use a measure of the distance between the correlation values (elements of the functional network) and the connection strength (elements of the structural network) we used *pdist* module with the *Euclidean* metric from Scipy package (Oliphant, 2007). This module calculates the average difference between the elements of the two matrices. More average distance means less similarity between the two matrices.

To calculate the distribution of correlations vs. weight and distance we binned structural data, i.e., we chose the links whose weight and distance lie in a bin around the given mean values. The width of the bins were set to 0.05 for the connection strengths and 16 mm for distances.

Finally, the communities were found based on the increasing mudulatory index using *community_walktrap* module from *Python-igraph* with steps = 4 (Pons and Latapy, 2005; Csardi and Nepusz, 2006).

### 2.1. Structural Network

**Figure 1:**
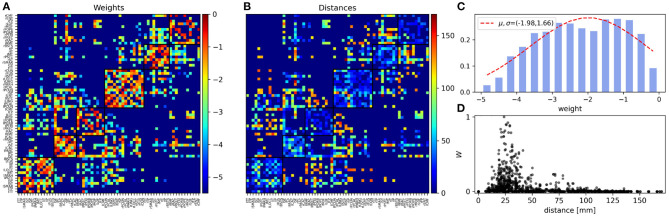
Structural properties of the human connectome. **(A)** The normalized coupling weights (0 ≤ *W* ≤ 1) and **(B)** Euclidean distances (in mm) in the human connectome with 66 nodes (Hagmann et al., 2008). The squares show the modules and the nodes ordered for the structural module (community) they comprise. The color bar of the weight matrix has a log_10_ scale. The background dark blue regions in A (< 10^−5^) and B (= 0) indicate the absence of edges between the areas. **(C)** Semi-log presentation of the distribution of the weights of structural connections that span five orders of magnitude. **(D)** Scatter plot shows the distribution of the normalized weights of the structural connections vs. the distance between the nodes. Here we have used a linear scale for both exes.

The matrix *A* was constructed based on the human connectome data with 66 nodes from (Hagmann et al., 2008). In this study, diffusion tensor imaging is used by applying six gradient directions, modeled the diffusion in each voxel as a sphere, and detected the amount of water diffusion. The main direction of water diffusion shows the regional white matter tracts. By connecting voxels based on their anisotropy and their principal diffusion direction, images of the major white matter pathways are constructed (Hagmann et al., 2008). In this method, the strength of the connection is determined by the average number of fibers between two regions. The structural properties of the Human connectome and distance matrix are shown in Figure 1.

We also used more recent data that has established interareal connectivity using sensitive retrograde tracers to determine the weighted connectivity of the inputs to 29 areas in an atlas of 91 cortical areas (Markov et al., 2014). Connection strengths have been derived from a connectivity matrix based on interareal connection strength in the macaque. The weight of a projection from a source area to a given target area is defined by the fraction of labeled neurons (FLN) expressed as the ratio between the number of labeled neurons in that source area over the total number of labeled cortical neurons extrinsic to the injected area. The dataset is available at cor-nets.org. For the simulations, the 29 × 29 directed graph *G*_29×29_ were used. *G*_29×29_ has *M* = 536 links out of the maximum possible of *N*(*N* − 1) = 812 with the density 66%. The structural properties of the macaque connectome and the distance matrix is shown in Supplementary Figure 1.

## 3. Results

In this paper, we aim to study the properties of the functional network of the brain at different frequency bands through simulation of a simple model of the human brain network. Specifically, we explore how the correlation between the oscillatory activity of the brain regions (nodes in the model) at different frequencies changes with the distance between the nodes. Our model is based on a generalized Kuramoto model run on top of the brain connectome composed of 66 nodes, whose properties are shown in Figure 1. Each node in the model is a phase oscillator which represents oscillatory dynamics of a region of interest of the brain in the given parcellation scheme (Hagmann et al., 2008). The frequency of the nodes is chosen from a narrow distribution around a mean value that is varied to represent the oscillatory dynamics of the brain regions over different frequency bands. The weights of the connections in the structural network, determined through diffusion MRI, are shown in Figure 1A, and the Euclidean distance between the nodes is shown in Figure 1B. The structural network shows a modular structure at two levels, with 6 modules at the first level and two modules at the second (corresponding to two hemispheres, see Model and Methods). Panel C depicts the histogram of weights which span five orders of magnitude. In Figure 1D we have shown the scatter plot of the connection strengths vs. the distance between the nodes, respectively. In particular, it is seen that most strong connections are distributed around short distances with 2 < *d* < 5 cm and distant nodes are connected by weak links. Although this effect could be an artifact of the tractography, other methods with more reliable measures for the connection weights, confirm that the weight of the connections between the brain regions decreases with distance (Ercsey-Ravasz et al., 2013; Donahue et al., 2016). For comparison, we have presented the properties of the structural network of the macaque with 29 nodes extracted from the tracing method in Supplementary Figure 1 and use this structural network to repeat all the subsequent simulations to show the generality of the results (Supplementary Figures 2, 3).

### 3.1. Frequency-Resolved Correlation Matrix

**Figure 2:**
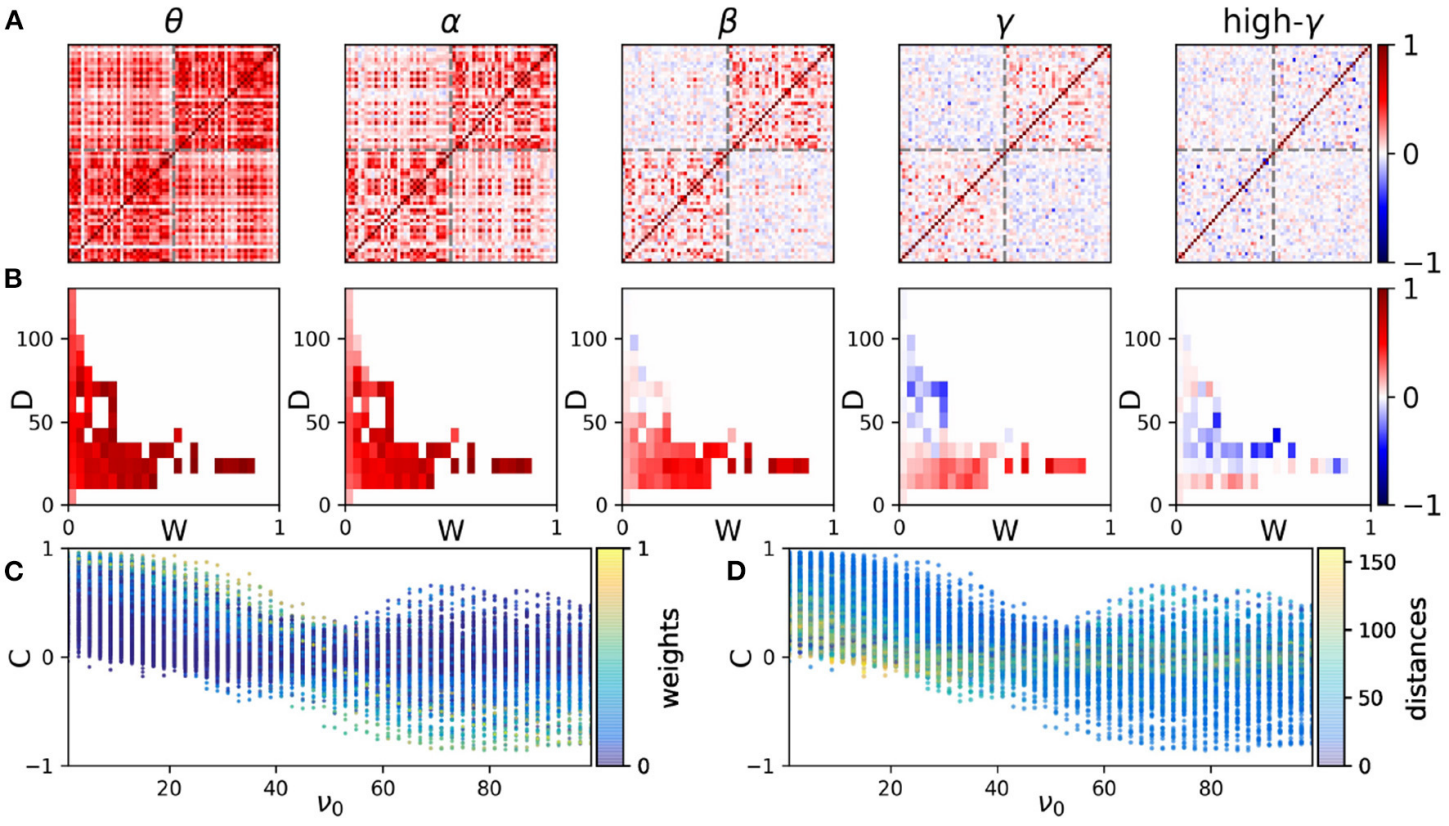
The correlation distributions. **(A)** The functional networks at five sample frequency ranges. The elements of the functional networks are the correlation indices σ_*ij*_ defined in the Methods. In each panel, the results of a simulation of the model with a given mean frequency are presented. From left to right the mean frequency is 3, 11, 23, 35, and 51 Hz which lie within the bands θ, α, β, γ, and high-γ, respectively. The frequencies are chosen from a normal distribution with the given mean value and standard deviation 0.1. The axes show the index of nodes which represent ROIs of the structural network. The gray dash lines indicate the boundary of the brain hemispheres. The coupling scale factor is *K*/*N* = 0.25 and the noise amplitude is 0.05. The initial phases are chosen from a uniform distribution in the range [−π, π]. The results are averaged over 200 realizations. **(B)** The distribution of correlations vs. weight (W) and distance (D) of the connections at each frequency. **(C,D)** The distribution of correlations vs. average natural frequencies of the nodes (ν_0_). The colors in panels **(C)** and **(D)** show the corresponding connection's weights and distances, respectively.

In the model, we assume that the interaction between the nodes takes place through a delay time which in general is dependent on the distance. The distribution of the delays turns out to be the determinant factor for the functional network at different frequency bands. We assume that the interaction delay is (linearly) proportional to the distance between the nodes, i.e., we take a fixed value for the speed of the signal transmission between the nodes (5 m/s) (Nakagawa et al., 2014; Petkoski and Jirsa, 2019). We also assume a weighted structural network where the connection strengths are scaled by the number of axonal tracts. The correlation matrix at five frequency ranges representing different frequency bands is shown in Figure 2A. The appearance of anti-correlation between some pairs of nodes over higher frequency bands is apparent. Anti-correlation first appears between the nodes with long-range connections in different hemispheres over the beta range (13–30 Hz), while in the gamma range (30–45 and 45–70 Hz for high gamma) they are also observed for shorter distances between the intra-hemisphere pairs. This indicates the possible role of distance in the correlation between the nodes at different frequencies.

The mean correlation between the nodes vs. connection strength and distance is shown in Figure 2B only for the pairs with a direct connection. At lower frequencies mean correlation shows no apparent conclusive dependence on the distance and connection strength. Anti-correlation appears at high distances at the beta range and shifts to lower distances with the increasing frequency following the results shown in Figure 2A. We note that by changing the value chosen for the signal transmission speed, the results change such that increasing the speed, anticorrelations appear at higher frequencies. However, the variations of correlation with distance are more apparent at higher frequencies regardless of the choice of the transmission speed.

To get insight into the role of transmission delays and connection strengths, we have shown scatter plots of the correlation index between all the pairs at different frequencies in Figures 2C,D, where colors indicate the weights (C) and distances (D) between the nodes, respectively. It is seen that the mean correlation between all the pairs of nodes decreases at higher frequencies and negative correlation is observed at higher frequencies for distant nodes. The left panel shows that strong connections (those with greater connection weight) lead to high positive and negative correlations at low and high frequencies, respectively. As it is seen in the right panel, at low frequencies the high positive correlation is seen mostly for low distances, while at higher frequencies short-distance nodes may show either positive or negative high degree of correlation. Long-distance nodes show lower values of correlation for all the frequencies. Similar results are obtained using Macaque connectome data (Markov et al., 2014) presented in Supplementary Figure 2. There it is confirmed that general results hold for a typical connectome network given that the connection strengths have a negative correlation with distance and the delays increase with the distance between the nodes. In the following, we inspect the frequency-resolved correlation matrix in more detail.

### 3.2. Relation to Distance and Frequency

**Figure 3:**
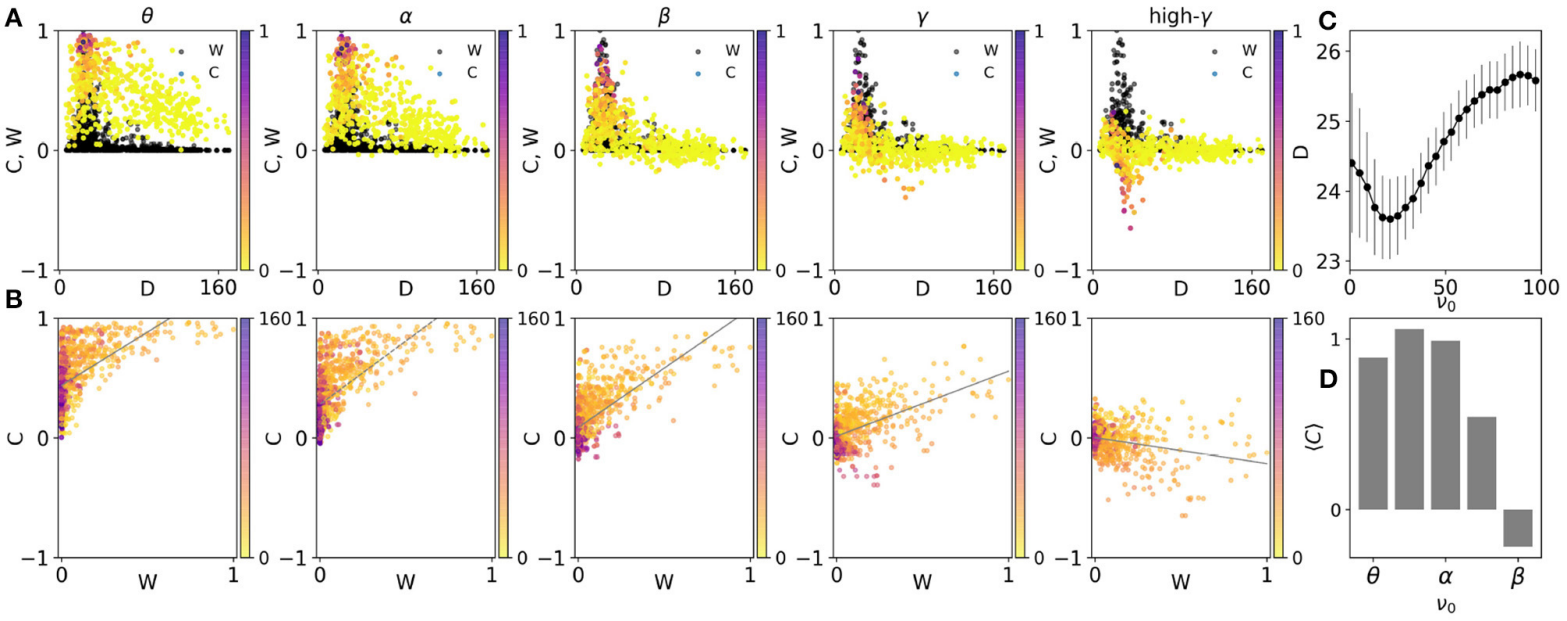
**(A)** The scatter plots of the average correlation between the nodes C, vs. distance at five average frequencies, 3, 11, 23, 35, and 51 Hz corresponding to theta, alpha, beta, gamma, and high gamma, respectively The colors indicate the corresponding weights of the structural connections. For comparison, the structural connections W, are also shown in black dots. **(B)** The scatter plots of the average correlations between the nodes vs. weights of structural connections. The colors indicate the corresponding distances at different frequencies. **(C)** The distance between scatter plots of the correlations and weights of connections in **(A)**. The inverse of this parameter is a measure of the similarity between the two matrices. **(D)** The slope of fitted lines in **(B)** vs. frequency.

We have shown the scatter plot of the correlation of the pairs vs. the distance of the nodes, at different frequencies in Figure 3A. We observe a negative correlation between the distance and correlation index, i.e., those nodes which are farther from each other have a lower correlation index. But, while at lower frequencies the correlation almost linearly decreases with distance, at higher frequencies a steeper drop with distance is observed similar to the structural distribution of the connection weights. As an important corollary, we have compared this distribution with the structural one (Figure 1D) by the distance measure introduced in Model and Methods. The best similarity between the distribution of structural and dynamical couplings between the nodes (which represent the structural and functional networks, respectively) characterized by the lowest distance between two distributions, is seen in beta and low gamma range around 30 Hz (Figure 3C). Similar results are obtained by using the macaque connectome shown in Supplementary Figure 3. We also note that the frequency range that shows the maximal similarity between the structural and the functional networks, depends on the choice of the speed of signal transmission. Notably increasing the speed of the signal transmission (decreasing delay) moves the frequency that shows maximum similarity to the higher frequency bands (see Supplementary Figure 3C).

We have also colored the points in Figure 3A based on the weight of the structural link between the nodes. It is observed that strong links overall lead to a larger pairwise correlation between the nodes, but this is only observable at low distances since there are hardly strong links between the far nodes (Figure 3D). Again, it is seen that while strong links lead to high positive correlation at low frequencies, they give rise to negative correlation at high-frequency ranges.

To more precisely inspect the relationship between the link strength and the correlation index between the nodes, we have shown them in the scatter plots of Figure 3B. Since in the connectome most of the links are very weak, the points in the scatter plot are packed in the small strength links. Nevertheless, the positive correlation between the link weight and correlation is observed for low frequencies and this correlation decreases with increasing frequency and turns to a negative correlation in high gamma range Figure 3D. Notably, the figure shows that very weak links can carry high correlations in low frequencies and short distances (shown by color). Similar results have been obtained on the macaque connectome shown in Supplementary Figures 3B,D.

### 3.3. Distinct Role of Connection Weight and Delay

Since both the connection strength and the delay in communication between the brain regions are dependent on the distance between them, we question what is their distinct role in the pairwise correlation? More specifically, previous results show that the distant nodes show a smaller correlation index at all frequency bands and they show anti-correlation for higher frequencies. Is that because they communicate through a longer delay or because they are connected by relatively weaker connections?

**Figure 4:**
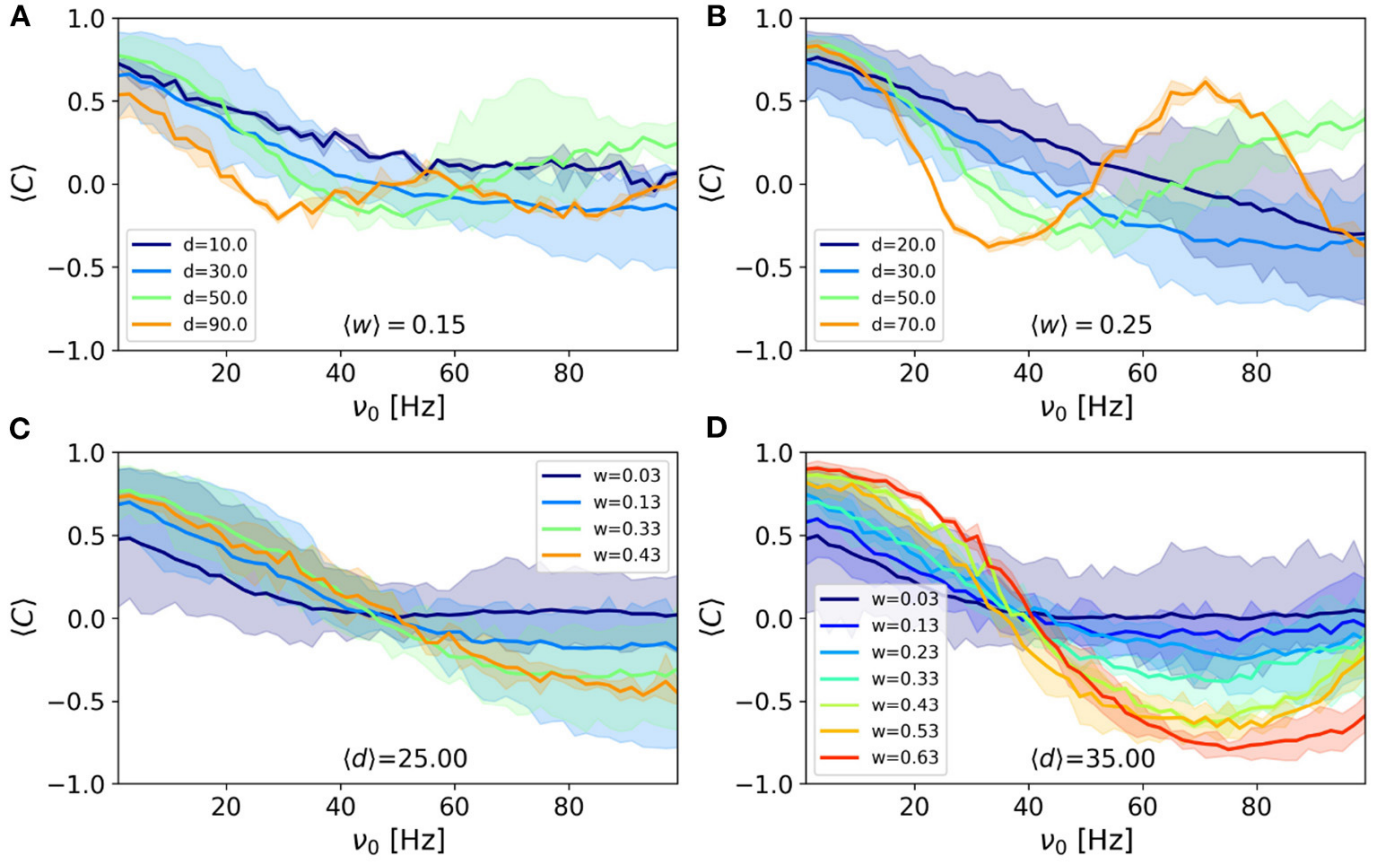
The average correlation vs. frequency for connections whose strength lies in the range **(A)** 〈*W*〉 = 0.15 and **(B)** 〈*W*〉 = 0.25 and various distances indicated in the legends. The average correlation vs. frequency for connections whose distance lies in the range **(C)** 〈*d*〉 = 25 mm and **(D)** 〈*d*〉 = 35 mm and various strengths indicated in the legends. Correlation is calculated using the correlation index σ_*ij*_ defined in Methods. The colored areas show the results for *p*-value = 0.05. The width of the bins was set to 0.05 for the connection strengths and 16 mm for distances.

To this end, we pick the pairs of nodes with almost the same connection strength locating at different distances. Note that fixing the connection strength, only the delay is changing when the distance is varied. We have shown the mean correlation vs. frequency for four different distances (delays) in Figures 4A,B (for two different strengths). The figure shows the values of the mean correlation index corresponding to the filtered edges at given delays and weights. It can be seen that the correlation shows an almost periodic behavior with frequency and the variation in correlation is faster for the pairs with a longer delay. A comparison of the two panels also shows that the amplitude of the changes is larger for stronger connections, while the rate of the change with frequency is only dependent on the communication delay and is independent of the connection strengths.

We also presented the results for the nodes which are at almost the same distance but are connected by different connection strengths. The results presented in Figures 4C,D (for two different distances) support the above results that stronger connections lead to a larger amplitude of variation while the pairs at the same distance, show the same rate of the change of correlation, with respect to frequency. Another point is that strong synapses not only give rise to higher correlation in low frequencies but also lead to more negative correlation at higher frequencies.

The presented results above show that the rate of the changes in the correlation with frequency is determined by the transmission delay but the amplitude of the variations is dependent on the link's strength. To confirm these results, we did two more simulation experiments by fixing the strength of connections or by fixing the delay in the interactions in the connectome. We first consider a binary structural network where the elements of the adjacency matrix *a*_*ij*_ are either zero or one and distance only affects the interaction delay. This can be considered also as an ultimate case where it is assumed that the connection strengths do not correlate with the distance. In this case, when binning the pair of nodes based on their distance is expected to lead to the curves seen in Figures 4A,B. The results shown in Supplementary Figure 4A conforms with this expectation and the nodes with different distances show different rates of change with respect to frequency. In the second experiment, we fixed the delay in the interaction between the nodes and retained the weight of the structural connection to those extracted from Figure 4A. We then binned the pair of the nodes based on the weight of their connections and inspected how the mean correlation changes with frequency. The results shown in Supplementary Figure 4B shows that the pairs with different structural weights show a periodic dependence on the frequency but the amplitude of the variation is dependent on the weight, right similar to Figures 4C,D.

## 4. Discussion

In this manuscript, we studied the dependence of the correlation between the oscillatory activities of the pair of nodes to their distance, at different frequency bands, through simulation of a system of delayed-coupled phase oscillators on top of the brain's connectome network. Since both delays in the communication between the nodes and the strength of the synaptic connections between them are a function of distance, we studied how the communication delay and connection strength can affect the correlation. We showed that the effect of these two parameters can be different at different frequencies. In particular, we found that at low frequencies the dependence of the pairwise correlation between the nodes is compatible with expectation and shorter delay and stronger connections lead to larger correlation. On the other hand at higher frequencies, the dependence is not trivial. Stronger connections in this range can lead to anti-correlation of the nodes and longer delays can both result in positive and negative correlation. In an intermediate-range, around beta and low gamma, we observed that the pattern of the correlations and the distribution of the weights against distance has maximal similarity to each other, compatible with the recent results (Ziaeemehr et al., 2020b).

In the studies on the synchronization of the oscillators on complex networks, the connection strength and the interaction delays are two determinant factors which their effect is extensively explored (Deco et al., 2009; Cabral et al., 2012; Wang et al., 2014; Petkoski et al., 2016; Asl et al., 2018; Madadi Asl et al., 2018). It is shown the phase relations between the pair of the coupled oscillators depend on the connection strength and to the delay (Yeung and Strogatz, 1999; Sadeghi and Valizadeh, 2014; Esfahani et al., 2016) and once the phase response function of the oscillators is known, the regions for the stability of in-phase, antiphase, and out of phase-locking can be determined (Esfahani and Valizadeh, 2014; Dumont and Gutkin, 2019). In the studies of large scale brain networks, the effect of conduction delay has attracted much interest in recent years and several studies have explored the effect of delay on the phase-locking between the oscillatory activity of the brain networks (Yeung and Strogatz, 1999; Deco et al., 2009; Lee et al., 2009; Petkoski et al., 2018; Petkoski and Jirsa, 2019). In particular, it has been shown that the appearance of in-phase and antiphase relation between the brain regions depends on the frequency, and antiphase locking is observed over higher frequency bands and for long-range connections with long delays (Petkoski et al., 2018; Petkoski and Jirsa, 2019). Our findings confirm these results although the parameters of the underlying structural networks, e.g., the distribution of the delays and connection strengths in Petkoski et al. (2018) were different from those we assumed. Beyond the effect of delay, however, our results suggest that the distance between the brain regions can affect the collective brain dynamics and the functional connectivity not only through delay but also through the distance-dependence of the structural connection's weight. One of the main focuses of the current study was to show that the delay and connection weights affect the frequency-dependent functional connectivity, in different ways.

One of our main assumptions in this study was the presence of a long tail distribution of the structural connection weights and a negative correlation between the weights and distance. We used two different datasets for the structural connectivity based on two different methods, tractography, and tracing, for identifying the connections between the brain regions. Previous studies in non-human primates demonstrate both successes and limitations of these two methods for assessing neurite trajectories (Jbabdi et al., 2015; Sotiropoulos and Zalesky, 2019). Of importance, none of the methods directly measure the synaptic strength and they only give indirect estimations for the weights of structural connections. Nonetheless, despite possible inaccuracies and pitfalls, both methods give qualitatively similar results on the wide distribution of the weights and the negative weight-distance correlation.

Brain functional networks are commonly constructed upon the linear statistical interdependencies between the activities of the brain regions which is conventionally measured by fMRI (Logothetis, 2008). The indirect measurement of the collective neuronal activity by fMRI can only reveal the slow dynamics of the brain due to its low temporal resolution, around 1 s (Kim et al., 1997; Sejnowski et al., 2014). Brain oscillations over several frequency bands which are known to be important for a variety of cognitive and executive functions have much shorter periods and it is impossible to assess them with BOLD signals. On the other hand, EEG and MEG recordings have a finer time resolution (Sejnowski et al., 2014; Burle et al., 2015). Recent instrumental advancements and the developments in data analysis software have made it possible to use EEG and MEG data to reveal the correlation between the brain networks' local dynamics in much finer time scales and a wide range of frequencies (Hillebrand et al., 2012; Gramfort et al., 2013). These warrant the need for theoretical and computational studies on the spectral properties of the correlation matrix and the functional networks.

Since these phase relations are hypothesized to underlie the communication between the brain populations, it is important to know how they change in realistic brain networks. In the brain networks, both the delay and connection strengths have a wide distribution making the brain structural network a very heterogeneous one. In this study, we used a realistic distribution for both the parameters and inspected how each of them impacts the pattern of the correlation between the brain regions, at different frequencies. With such a wide distribution of these parameters, a diversity of the correlations and the phase relations are observed which are important for a diverse and dynamic communication pattern in the brain (Ghosh et al., 2008; Breakspear et al., 2010; Maris et al., 2016).

While we did not directly explore the phase difference between the activities of the nodes, changes in the correlation could indirectly determine the phase relations. Namely, a high positive and negative correlation could indicate an almost in-phase or antiphase evolution of phases, respectively, with a continuum of intermediate phase differences between the two extremes. Our results indicated that the phase relations for any pair of nodes are in general dependent on the frequency. This has an important functional implication for the communication between the brain's areas. Since the phase differences could determine the effective functional connectivity between the nodes (Friston, 2011; Maris et al., 2016), the pairs can communicate at different frequencies with different efficacy at multiple frequency bands. Such a multiplex of effective functional networks makes it possible to simultaneously engage the nodes at multiple functional modules (Park and Friston, 2013).

Moreover, our results showed more diverse phase relations at higher frequencies. Indeed over low-frequency bands, the correlation more slowly changes with distance and this means that long-range communication between the brain areas can take place by slow dynamics. On the other hand, a faster change in correlation with distance at high frequencies makes it possible to functionally dissociate the areas at a certain distance and form local functional modules. This can be a fundamental need for the brain networks for segregation of information processing at high-frequency bands and global integration at low frequencies (Isomura et al., 2006; Buzsáki and Mizuseki, 2014). The presence of multiple frequency bands could then lead to a hierarchy of spatial scales over which the information is integrated, corresponding to each frequency band (Zhou et al., 2006; Meunier et al., 2010). Our results show that the heterogeneous communication delay is the key requisite for the brain to enable such a hierarchical integration of information.

## Data Availability Statement

The source code for the simulations and reproducing the figures of the manuscript are available through the following link: github.com/Ziaeemehr/Frontiers2021.

## Author Contributions

AV designed the research. AZ performed the numerical experiments. AZ and AV analyzed the data and wrote the paper. Both authors contributed to the article and approved the submitted version.

## Conflict of Interest

The authors declare that the research was conducted in the absence of any commercial or financial relationships that could be construed as a potential conflict of interest.
