# Supplementary material for: Frequency-Resolved Functional Connectivity: Role of Delay and the Strength of Connections
Source: Front Neural Circuits. 2021 Mar 24;15:608655. doi: 10.3389/fncir.2021.608655 (PMC8024621; doi:10.3389/fncir.2021.608655)
Supplement: Supplementary file 1 [file Data_Sheet_1.PDF]

Supplementary information: Frequency-resolved functional connectivity: Role of delay and the strength of connections

Abolfazl Ziaeemehr[1] and Alireza Valizadeh[1,2]

[1]Department of Physics, Institute of Advanced Studies in Basic Sciences (IASBS), Zanjan, Iran.
[2]School of Biological Sciences, Institute for Research in Fundamental Sciences (IPM), Tehran, Iran.

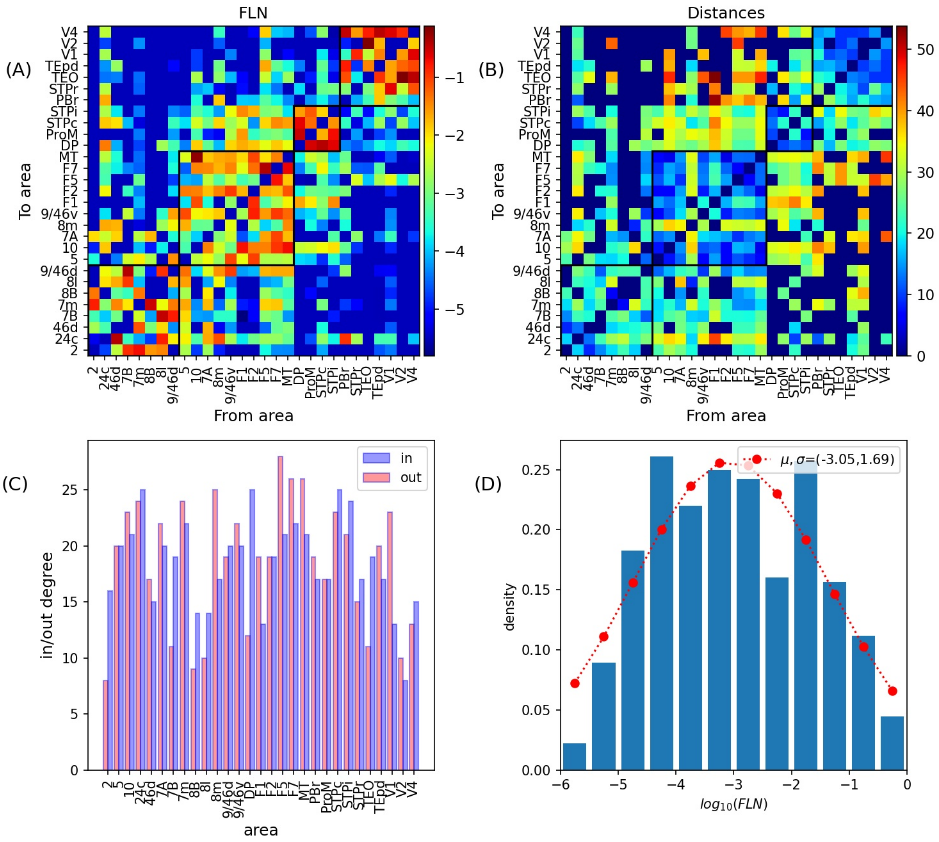

Figure S1: **The structural properties of the macaque connectome.** (A) The FLN values with $\log_{10}$ scale color bar and (B) The Euclidean distances (in mm) in the macaque connectome with 29 nodes (Markov et al., 2014). The solid squares show the modules and the nodes reordered for their communities. The background blue regions in A ($< 10^{-5}$) and B ($= 0$) indicate the absence of edges between the areas. (C) In and out-degree of each node. (D) The weights of connections span six orders of magnitude and follow a lognormal distribution in the density plot.

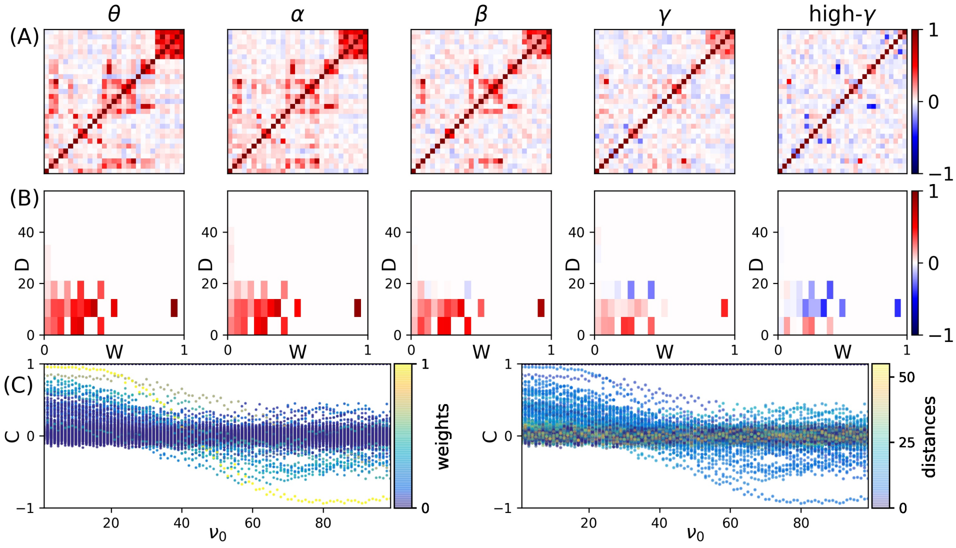

Figure S2: **The correlation distributions for directed connectome of the macaque.** (A) The correlation matrices at five different average frequencies of the nodes, 3, 11, 23, 35, and 51 Hz corresponding to $\theta, \alpha, \beta, \gamma$, and high-$\gamma$, respectively. The axes show the index of nodes which represent ROIs of the structural network. The overall coupling strength is $K/N = 0.3.$, The frequencies have a normal distribution with a standard deviation $SD = 0.1$ and the amplitude of the noise $D = 0.05$. The initial phases have a uniform distribution in $[-\pi, \pi]$. The results averaged over 150 realizations. (B) The distribution of correlations versus weight (W) and distance (D) of connections at each frequency. (C) The distribution of correlations versus average natural frequencies of the nodes (ν_0). The colors in left and right panels show the corresponding weights and distances, respectively.

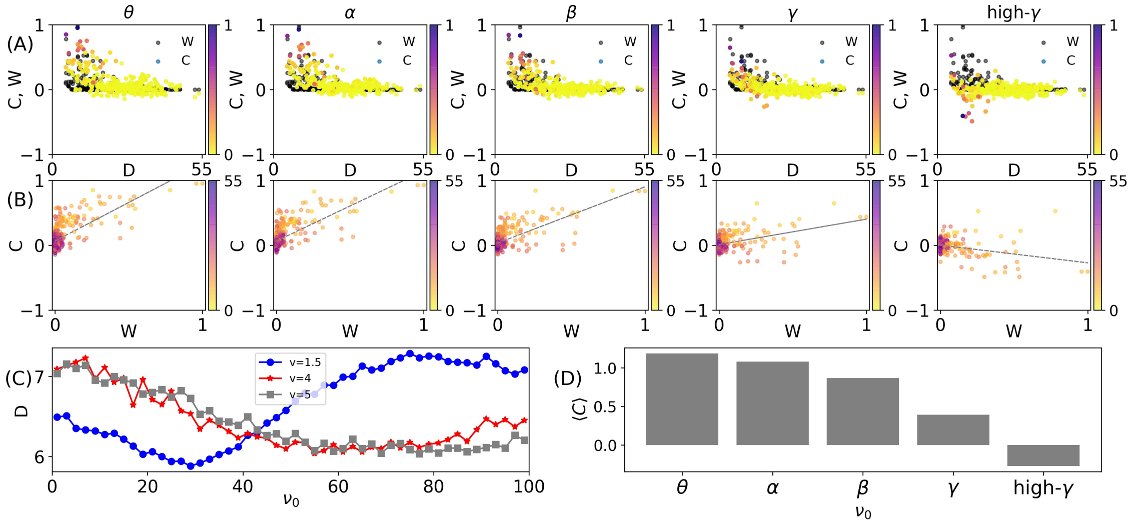

Figure S3: **Comparison of correlation and connection weights distribution for the directed connectome of the macaque.** (A) The scatter plots of the average correlation matrices versus distance at five average frequencies, 3, 11, 23, 35, and 51 Hz corresponding to $\theta, \alpha, \beta, \gamma$ and high-γ, respectively The colors indicate the corresponding weights of the connections. For comparison, the structural connections W, are also shown in black dots. The overall coupling strength $K/N = 0.3$, variance of initial frequencies $SD = 0.1$ and the amplitude of noise $D = 0.05$. (B) The scatter plots of the average correlations matrices versus weights of connections. The colors indicate the corresponding distances at different frequencies. (C) The Euclidean distance between scatter plots of correlations and weights of connections for different signal transmission velocity. (D) The slope of fitted lines in panel (B) versus frequency. The results averaged over 150 realizations and the parameters of the simulation is similar to the figure S2.

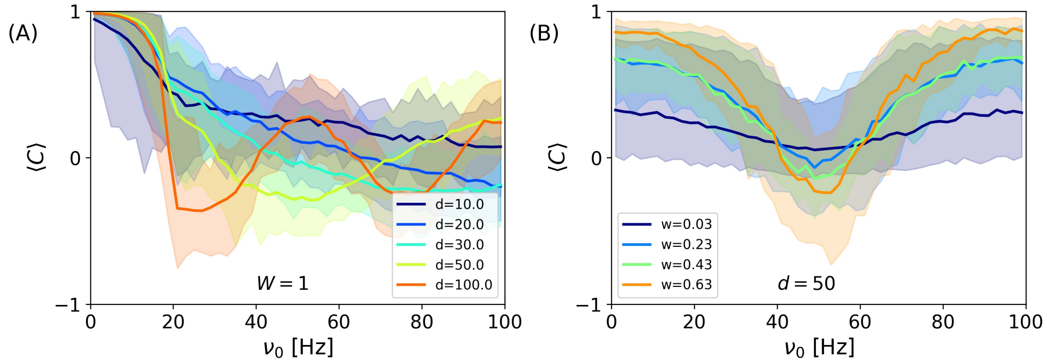

Figure S4: **(A)** The average correlation versus frequency for the binary human connectome and different distances in the legend. The overall connection strength $K/N = 0.01$ **(B)** The average correlation versus frequency for the human connectome with constant delay = 10 ms (distance = 50 mm) and different weights of connections in the legend. The overall connection strength $K/N = 0.3$. The amplitude of noise $D = 0.05$ and variance of initial angular frequencies $SD = 0.1$. The colored areas show the results for $p\text{-}value = 0.05$. The width of the bins was set to 0.05 for the connection strengths and 16 mm for distances.

References

Markov, N. T., Ercsey-Ravasz, M. M., Ribeiro Gomes, A., Lamy, C., Magrou, L., Vezoli, J., Misery, P., Falchier, A., Quilodran, R., Gariel, M.-A., et al. A weighted and directed interareal connectivity matrix for macaque cerebral cortex. *Cerebral cortex*, 24(1):17–36, 2014.
